# Microfluidic Multitissue Platform for Advanced Embryotoxicity Testing In Vitro

**DOI:** 10.1002/advs.201900294

**Published:** 2019-04-29

**Authors:** Julia Alicia Boos, Patrick Mark Misun, Astrid Michlmayr, Andreas Hierlemann, Olivier Frey

**Affiliations:** ^1^ Bioengineering Laboratory Department of Biosystems Science and Engineering ETH Zürich Mattenstrasse 26 4058 Basel Switzerland; ^2^ InSphero AG Wagistrasse 27 8952 Schlieren Switzerland

**Keywords:** 3D microtissues, body‐on‐a‐chip, developmental toxicity, embryonic stem cell tests, microfluidics

## Abstract

The integration of metabolic competence in developmental toxicity assays in vitro is of fundamental importance to better predict adverse drug effects. Here, a microfluidic hanging‐drop platform is presented that seamlessly integrates liver metabolism into the embryonic stem cell test (EST). Primary human liver microtissues (hLiMTs) and embryoid bodies (EBs) are combined in the same fluidic network, so that hLiMT‐generated metabolites are directly transported to the EBs. Gravity‐driven flow through the network enables continuous intertissue communication, constant medium turnover, and, most importantly, immediate exchange of metabolites. As a proof of concept, the prodrug cyclophosphamide is investigated and a fourfold lower ID50 concentration (50% inhibition of EB differentiation) is found after biotransformation, which demonstrates the potentially adverse effects of metabolites on embryotoxicity. The metaEST platform provides a promising tool to increase the predictive power of the current EST assay by more comprehensively including and better reflecting physiological processes in in vitro tests.

## 1 Introduction

Developmental and reproductive toxicology (DART) tests constitute a central part of every drug development process.^1^ Every year, 5–7% of newborns worldwide are born with serious birth defects,^2^ a number that is correlated to an increasing consumption of pharmaceuticals for managing health conditions.^3^ To date, toxicological safety assessment of new pharmaceuticals is still conducted on animals in vivo, about 90% of which are related to reproductive toxicology.^4^ According to FDA standards, these animal studies need to be performed in two species: a primary rodent species and a secondary mammalian species, mostly rabbits. These studies not only include male and female animals but also their offspring and fetuses.^5^, ^6^ These tests are not only ethically disputed but also lack physiological relevance, as data cannot be directly translated to human conditions.^7^ Moreover, the high costs of DART testing entail that these studies are performed at a very late stage in preclinical trials. In view of increasingly stringent safety regulations, such as REACH,^8^ and initiatives for alternatives to animal testing (3Rs^9^), the demands of DART testing have become a tremendous challenge.

In addressing this challenge, substantial work has been invested in developmental in vitro assays as cost‐ and time‐efficient alternatives to animal tests.^10^ Among three validated assays,^11^, ^12^, ^13^ the embryonic stem cell test (EST^14^) is the only in vitro system that entirely relies on the use of a cell line. The EST is used to assess the embryotoxicity of compounds by employing the spontaneous differentiation of murine embryonic stem cells (mESCs) as a model for early embryonic development. In this test, 3D spherical embryoid bodies (EBs) are formed from pluripotent mESCs in the presence of a compound and serve as complex cell model to study spontaneous cell differentiation. The readout is based on the potential of the mESCs to differentiate into contracting cardiomyocytes, which is morphologically assessed by microscopy analysis. The embryotoxic potency of a compound is determined by evaluating the concentration, which leads to 50% inhibition of differentiation (ID50) and the corresponding impaired embryonic development.

In industry, the EST is currently applied as a screening method in tiered test strategies to preselect potential drug candidates prior to animal tests, but it does not yet provide a viable alternative to animal testing.^1^, ^15^ Despite its high predictive power of 78% accuracy relative to in vivo studies,^16^ drug‐metabolism effects are not included in the assay, what is a major reason for its limited predictive power and consequently prevents the full replacement of animal models.^17^ The current assay only provides information on the direct toxicity of a test compound, neglecting metabolic processes and interactions between organs. A significant part of pharmaceutical compounds, however, require metabolic activation, for example in the liver, in order to exert toxic effects on target cells or tissues.^18^, ^19^ Consequently, metabolic effects constitute an indispensable part of toxicological safety assessment and are pivotal for determining embryotoxic effects.

Several in vitro methods that include metabolism have been developed by adding the liver as the predominant organ in biotransformation.^20^, ^21^ The incorporation of metabolic capacity into developmental toxicity assays however, proved to be challenging. Biotransformation directly in the culture by adding subcellular fractions, such as microsomes, obtained from liver homogenates, was found to be too cytotoxic to be used in long‐term toxicity studies.^22^ More promising approaches included static coculture systems of hepatocytes in 2D cultures or suspensions of hepatocytes together with embryonic tissue^23^, ^24^ as well as systems that rely on preincubation of compounds with hepatocytes.^25^ The latter strategy has also been applied in the EST.^26^, ^27^ Nevertheless, the currently used systems still lack important features of the in vivo situation to closely reproduce physiological conditions.

First, biologically more relevant liver models are needed. Such models can be obtained from liver slices^28^ or isolated perfused livers,^29^ which are, however, not suitable for long‐term developmental toxicity studies due to their short life‐span in vitro. Growing cells in 3D cell constructs offers a more reproducible alternative also for long‐term toxicity studies.^30^, ^31^ Such 3D microtissues can be formed from various cell types and display highly organotypic functions, therefore providing a suitable model for clinically relevant in vitro studies.^32^, ^33^ Second, static and well‐plate‐based culture systems without flow do not include all components that are important for a systemic understanding, as they do not include intertissue communication and the exchange of unstable and short‐lived metabolites. A continuous liver‐compound‐embryo interaction is, therefore, crucial for the extraction of physiologically relevant information.

Microfluidic body‐on‐a‐chip approaches have been demonstrated to offer great potential to develop new assays and make them more representative. Advanced and more relevant in vitro platforms have been designed that are capable of better mimicking physiological processes on a microscale.^34^, ^35^ Several organ models have been cultured in one fluidic network,^36^ in which interconnection between the tissues was enabled through microchannels. The integration of 3D cell culture models in such perfused platforms featuring intertissue interaction and communication further increases their physiological relevance,^37^ while the platform technology allows for precise control of system parameters and enables microscopy observation.^38^

Here, we present a microfluidic multitissue platform to seamlessly integrate liver metabolism into the EST. In the “metaEST,” we exploit the hanging‐drop‐network (HDN^39^) technology as microphysiological system to form EBs on‐chip and coculture them with primary 3D human liver microtissues (hLiMTs) in immediate proximity to each other. The entire metaEST assay is performed in a single microfluidic device, which is operated by gravity‐driven flow to ensure constant intertissue communication and rapid and efficient exchange of metabolites. As a proof of concept study, we investigated the activation of the prodrug cyclophosphamide (CP), which needs metabolic conversion to produce teratogenic effects.

## 2 Results

### 2.1 Realization of the metaEST

**Figure 1:**
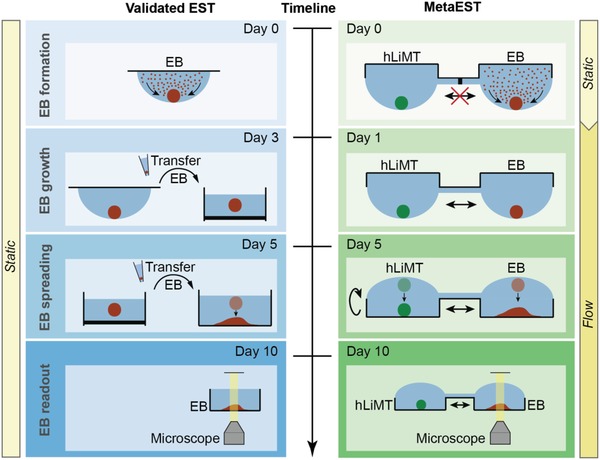
Concept of metaEST. Side‐by side comparison of the metaEST concept and the validated standard EST. EBs (red) from mouse embryonic stem cells are formed at the liquid–air interface of a hanging drop under static conditions. During EB formation, the EB compartment of the metaEST is separated from the compartment hosting human liver microtissues (hLiMTs, green), both of which are on the same chip. At day 1 of the metaEST, hLiMTs and EBs are fluidically interconnected in the same network, while the validated EST is continued under static and monoculture conditions. In the EST, the EBs are then transferred to suspension culture on day 3 to replenish the medium. In the metaEST, this transfer is not necessary, as medium is exchanged directly on‐chip. At day 5, the EBs in the validated EST are manually transferred to an adhesive surface, where they adhere and spread out. In the metaEST this step is realized by flipping the microfluidic chip upside down to a standing‐drop configuration so that the substrate of the chip serves as a substrate for EB spreading. At day 10, the EBs are morphologically inspected under the microscope to detect beating areas in the spread tissues.

The overall concept of the microfluidic metaEST is schematically shown in **Figure** **1** and compared side‐by‐side to the protocol of the commonly used and validated conventional EST. Both assays begin with the formation of embryoid bodies from a suspension of mouse embryonic stem cells at the liquid–air interface of a hanging drop. The validated EST is performed in its entirety under static and monoculture conditions (left‐hand side of Figure 1). After 3 d of tissue formation, EBs are transferred into a suspension culture to replenish the medium and they are then grown in suspension for two more days. On day 5, individual EBs are manually transferred to a third substrate, a well plate with a bottom surface that allows for tissue adhesion and spreading. This step is necessary to get optical access to contracting areas within the EBs, the activity of which is used as a readout on day 10 to evaluate embryotoxic effects of the compounds under test.

In contrast, we performed the entire metaEST on a single microfluidic platform in coculture with hLiMTs (right‐hand side of Figure 1). During EB formation, preformed hLiMTs were cultured in disconnected hanging drops under static conditions. We established fluidic interconnection between the drops hosting the microtissues (MTs) after 24 h, in order to connect hLiMTs and EBs within the same network. From that point on, liquid exchange between the compartments enabled direct intertissue communication, continuous medium turnover and, most importantly, dosage of target compounds and exchange of their metabolites. Medium exchanges throughout the assay were executed directly on‐chip. On day 5, we flipped the microfluidic chip upside‐down to attain a standing‐drop configuration. The MTs settled to the substrate of the chip, and the EBs started to adhere and spread on the adhesive surface. The surface below the hLiMTs was nonadhesive so that the hLiMTs preserved their morphology and function. At day 10 of the assay, we optically assessed the EBs for cardiomyocyte differentiation directly on the microfluidic platform by searching for contracting areas in the spread‐out tissues. We included metabolic competence in the EST by establishing a coculture and interaction of EBs and hLiMTs through the liquid phase in a controlled culturing environment. Moreover, we integrated the metaEST in one microfluidic platform and reduced the experimental complexity in comparison to the three‐step protocol of the validated standard assay.

### 2.2 Establishing a Microfluidic metaEST

**Figure 2:**
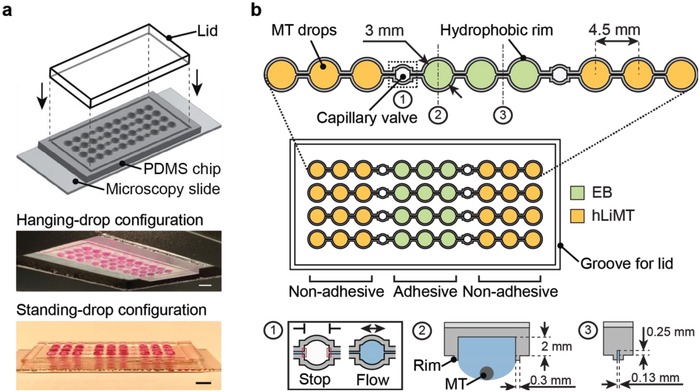
Microfluidic multitissue hanging‐drop platform. a) The microfluidic network is patterned on the surface of a PDMS substrate. The substrate is bonded to a microscopy slide, and a lid is inserted into a groove structure to cover the open system. Photographs of the chip show its operation in hanging‐drop and standing‐drop configuration. Scale bar: 5 mm. b) The chip layout consists of four individual lanes of nine interconnected drops. A hydrophobic rim structure of 0.3 mm is surrounding the liquid compartments and connecting channels of each lane and defines the liquid boundaries. Three drop structures form subunits at a pitch of 4.5 mm, which are separated by capillary valves. Inset (1) shows a capillary valve in its closed (stop) and open (flow) state. Each drop serves as culture compartment for one microtissue (MT, inset 2). The compartment has a diameter of 3 mm and a recess of 2 mm. The drops are fluidically interconnected through microchannels of 0.13 mm diameter and 0.25 mm depth (3). The EBs are cultured in the central compartments (green), which are coated adhesively. The hLiMTs are cultured in the neighboring compartments at the left and right (yellow) featuring a nonadhesive coating.

We realized the metaEST by devising a multitissue configuration in a microfluidic HDN, which features an ideal environment for the formation and cocultivation of 3D spherical microtissues. **Figure** **2** shows the layout and design of the microfluidic hanging‐drop platform. The microfluidic chip consisted of a surface‐patterned polydimethylsiloxane (PDMS) substrate, which was bonded to a microscopy slide for improved stability and planarity (Figure 2a). The patterns of the PDMS substrate served as liquid‐phase guides to form a completely open microfluidic system with circular hanging‐drop structures and interconnecting channels. A lid was inserted into a rectangular groove structure to cover the open microfluidic network and limit evaporation. The chip was operated in a hanging‐drop and standing‐drop configuration.

The chip consisted of four individual lanes comprising nine interconnected drops each (Figure 2b). The microfluidic lanes were defined through hydrophobic rim structures that prevented liquid overflow and enabled stable operation. Every lane included three subunits of three drops at a pitch of 4.5 mm, which were separated from the neighboring subunits by capillary‐stop valves. Each drop compartment had a diameter of 3 mm and included a well with a recess of 2 mm depth to further increase the medium volume. Each drop served as a culture compartment for a single microtissue. In order to avoid MT dislocation to neighboring compartments, the interconnecting microchannels were designed with a diameter of 130 µm, which was less than the diameter of the MTs.

Capillary stop valves allowed for temporary separation of hLiMT‐ and EB‐compartments, which ensured an mESC seeding and EB formation in the central subunit (Figure 2b, green) while obviating liquid or cell spill‐over into the neighboring drops hosting the hLiMTs (Figure 2b, yellow). The EB and MT subunits were afterward interconnected by wetting the capillary valves with medium and establishing a fluidic coculture network. hLiMTs were cultured adjacent to EBs in neighboring drops at the left and right side to attain immediate and homogenous exposure of the EBs to liver‐generated metabolites.

An adhesive surface coating was used to promote the spreading of the EB in the standing‐drop configuration, while a nonadhesive coating was used in the hLiMT compartments to maintain the compact 3D morphology and functionality of the hLiMTs during the whole assay.

**Figure 3:**
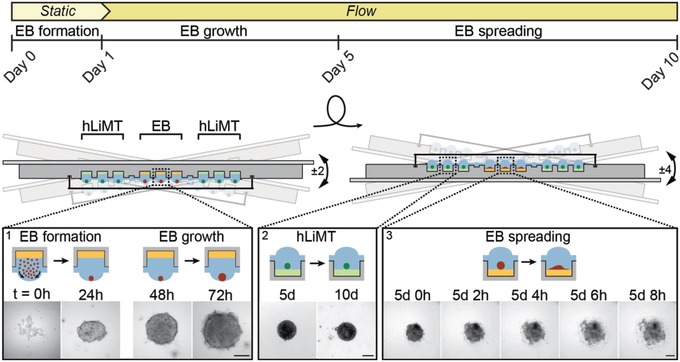
Schematic of the microfluidic metaEST. The timeline including applied flow conditions and EB culture phase is shown at the top, while the operation of the platform is shown below. During the first 5 d, the chip is operated in a hanging‐drop configuration for embryoid‐body (EB) formation and growth (inset 1, *t* = 0–72 h). EBs are formed during the first day from a cell suspension under static conditions (static). Preformed human liver microtissues (hLiMTs) are cocultured in adjacent drops from the beginning of the assay. At day 1, gravity‐driven flow through the platform is induced by tilting the chip ±2°, so that intertissue communication through the liquid phase is established. At day 5, the platform is flipped upside down to a standing‐drop configuration and tilted by ±4°. In this configuration, hLiMTs and EBs settle to the bottom of the wells. EBs start spreading (inset 3, *t* = 5 d 0 h to 5 d 8 h) on the substrate, which is promoted by an adhesive coating (yellow). hLiMT compartments are coated nonadhesively (green) to maintain spherical morphology (inset 2, day 5 and day 10) of the microtissues. The microscopy‐based assessment is carried out at day 10. Scale bar: 200 µm.

The implementation and execution of the metaEST within a microfluidic HDN is shown in **Figure** **3**. During the first 5 d of the assay, we operated the chip in a hanging‐drop configuration for EB formation and growth. In this configuration, mESCs sedimented at the liquid–air interface of the hanging drops and formed spherical EBs, which were differentiating and growing (Figure 3, inset 1). The platform was kept in a horizontal position during the first 24 h to ensure a homogeneous distribution of the mESCs after cell seeding and to guarantee an undisturbed EB formation under static conditions. At the same time, preformed hLiMTs were cultured in adjacent drops that remained yet disconnected from the EBs. After establishing coculture conditions by opening the capillary valves at day 1, gravity‐driven flow through the network was induced by slight tilting of the chip by ±2° every 15 s. Tilting by 2° allowed for stable operation of the system with an average medium turnover of 4.5 µL per tilting cycle. Tilting enabled a quick medium exchange between the compartments, i.e., direct intertissue communication through signaling molecules, continuous transport of compounds and exchange of metabolites. Due to the proximity of the MTs in the neighboring compartments and the rapid liquid‐phase exchange, the effects of transiently stable and highly reactive intermediates could be examined. After 5 d, we flipped the chip upside down to a standing‐drop configuration and continued tilting at ± 4° every 15 s with an average medium turnover of 8.7 µL per tilting cycle. In contrast to a hanging‐drop configuration, stable operation at larger tilting angles was possible in the standing‐drop regime, as gravity did not act against the drop's surface tension anymore. hLiMTs and EBs settled to the bottom of the well in the compartments, which helped to avoid high shear forces upon tilting. EBs started spreading out on the surface of the culture compartments, supported by the adhesive coating. Spreading continued within the first hours after flipping of the chip, and the EBs grew and differentiated on the surface (Figure 3, inset 3). In contrast, the nonadhesive surface coating in the liver compartments helped to maintain the compact and spherical shape of the hLiMTs (Figure 3, inset 2). The platform was operated in the standing‐drop configuration for another 5 d, before the EBs were optically inspected for contracting cardiomyocytes. The open microfluidic chip design enabled easy access to the MTs for harvesting and further downstream analysis.

### 2.3 Coculture Characterization of EBs and hLiMTs

The successful coculturing of EBs and hLiMTs greatly depended on finding a suitable medium that helped to maintain viability and functionality of both tissue models over the whole duration of the assay. The formation of the EBs and proper differentiation into contracting cardiac tissue relies on several factors that are commonly found in fetal bovine serum (FBS). In contrast, liver microtissues are commonly cultured under serum‐free conditions. We tested different media compositions with varying concentrations of FBS and additional culture supplements, shown in Table S1 (Supporting Information). A successful cocultivation of EBs and hLiMTs could be achieved in a tailored ‘metaEST medium' of advanced Dulbecco's Modified Eagle Medium (DMEM), enriched with 5% (v/v) FBS, 1% (v/v) nonessential amino acids, 1% (v/v) 2‐mercaptoethanol, 1% (v/v) penicillin/streptomycin, and 3.5% (v/v) primary hepatocyte maintenance supplement.

**Figure 4:**
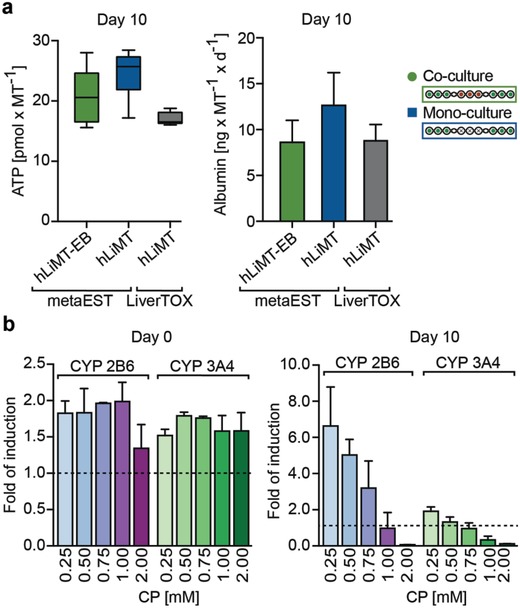
Functional characterization of human liver microtissues (hLiMTs). a) ATP content and Albumin production under coculture and monoculture conditions were evaluated at day 10 in the metaEST medium and compared to monoculture conditions in hLiMT‐specific control medium “Liver TOX” (InSphero, Switzerland). All culture drops were connected under coculture and monoculture conditions. Mean ± SD, *n* = 5–9 hLiMTs. b) CYP activity of CYP2B6 and CYP3A4 enzymes at day 0 and day 10 upon induction with CP in a dose‐range of 0–2 × 10^−3^ m. hLiMTs were induced with CP 24 h prior to day 0 and constantly exposed to CP during the assay. Data were normalized to basal activity (dotted line) at 0 × 10^−3^ m CP. For absolute values see Figure S1 (Supporting Information). Mean ± SD, *n* = 3 hLiMTs per condition.

After having defined the medium, first, we characterized hLiMT viability and the status of liver‐specific functions over the whole duration of the assay (**Figure** **4**). We tested viability and functionality in the metaEST medium under coculture and monoculture conditions and compared the obtained results to measurement values in liver‐specific control medium (Liver TOX), shown in Figure 4a. Under both conditions, all culture drops were connected, whereas, under monoculture conditions, the central compartments were filled with medium and did not host EBs. Primary hLiMTs retained viability for at least 10 d in the metaEST system, as demonstrated by measuring the intracellular ATP content. We observed elevated ATP values of EBs in the metaEST medium with 20.6 ± 4.7 pmol MT^−1^ (coculture) and 24.6 ± 4.0 pmol MT^−1^ (monoculture) in comparison to the control values of 16.0 ± 3.12 pmol MT^−1^. These increased ATP values can be explained by the presence of FBS and linoleic acid in the metaEST medium, which constitute an additional energy source and a supply of trophic factors that support growth and metabolic activity of the tissue. In contrast, the LiverTOX control medium consists of a minimal medium composition without any additional proteins. Moreover, we evaluated the secretion of the hepatocyte‐specific marker albumin to assess the functionality of the hLiMTs. The hLiMTs remained functional in the metaEST medium over 10 d with an average albumin secretion of 8.6 ± 2.4 and 12.6 ± 3.6 ng MT^−1^ d^−1^ under coculture and monoculture conditions. These albumin levels were comparable to those obtained with control medium: 8.8 ± 1.8 ng MT^−1^ d^−1^.

As we used the compound CP, a prodrug with known embryotoxic effects,^40^ in a metaEST proof of concept experiment, we characterized the metabolic activity of the hLiMTs through assessing the activity of cytochrome P450 (CYP) enzymes 2B6 and 3A4, the predominant isoforms involved in metabolizing CP (Figure 4b and Figure S1, Supporting Information).^41^ We measured CYP activity at day 0 and day 10 of the assay upon induction with CP in a dose‐range between 0 × 10^−3^ and 2 × 10^−3^ m. hLiMTs were induced with CP 24 h prior to the experiment and constantly exposed to the drug during the assay. At day 0 (24 h after CP induction), up to twofold elevated metabolic activity was measured in the case of CP induction in comparison to basal levels. At day 10, a sevenfold increase of CYP2B6 and a twofold increase of CYP3A4 activity was observed for 0.25 × 10^−3^ m CP. CYP activity decreased with increasing overall CP dose as a consequence of the hepatotoxic effects of CP on liver functionality over time. The viability of the hLiMTs remained constant up to a dose of 1 × 10^−3^ m CP (Figure S2, Supporting Information).

**Figure 5:**
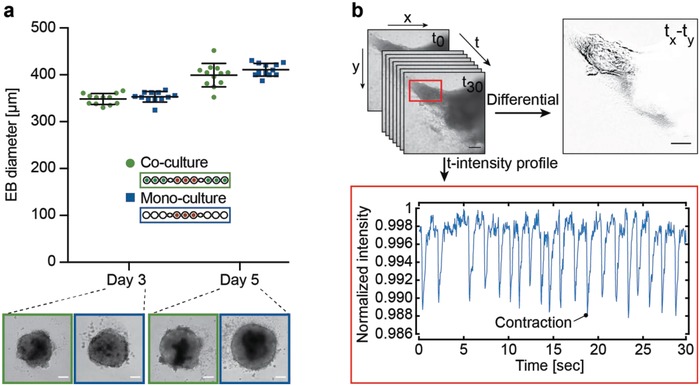
Functional characterization of embryoid‐body (EB) differentiation. a) Measurements of EB diameters on chip under coculture (green) and monoculture (blue) conditions at day 3 and 5; mean ± SD, *n* = 12 EBs per condition. Representative bright‐field images are shown for each condition; scale bar: 100 µm. b) Functional readout of beating cardiomyocytes. An example beating trace of an EB at day 10 was captured by recording during 30 s (Movie S2, Supporting Information). A differential image was generated through image subtraction of two subsequent images at contraction and relaxation ( *t_x_*–*t_y_*). The intensity profile of mean gray values of every time‐lapse acquisition in the area of contraction evidences the beating intervals and frequency during 30 s; scale bar: 200 µm.

In a next step, we verified the differentiation capacity of the EBs that is used to assess healthy or compromised embryonic development (**Figure** **5**). EBs, homogeneous in size, were reproducibly formed on‐chip as shown in Figure 5a and Movie S1 (Supporting Information). At day 3, we measured mean EB diameters of 349 ± 12 µm under coculture conditions and of 353 ± 11 µm under monoculture conditions. At day 5, we obtained EB diameters of 400 ± 25 µm under coculture conditions and 411 ± 14 µm under monoculture conditions.

Then, we assessed the influence of liquid flow on the spreading behavior and differentiation potential of the EBs in the absence of hLiMTs. We observed beating areas in all the microtissues under flow conditions as well as a mean delay of ≈40 h in the onset of spreading in comparison to static conditions (Figures S3 and S4, Movies S2 and S3, Supporting Information). This difference can be explained by small movements of the microtissues, which are caused by the liquid flow and consequently delayed tissue adhesion to the surface.

The functional readout for EB differentiation capacity on day 10 included to visually assess the presence or absence (yes/no criterion) of contracting cardiomyocytes within the tissue outgrowth. Figure 5b shows an exemplary beating area within a spread EB, which yielded a positive (yes) readout result. The beating areas in the flattened EBs were assessed with the microscope through continuous image acquisition during 30 s at a frame rate of 24 fps. The presence of beating activity was then assessed by means of differential images, obtained through subtraction of two images taken at contraction and relaxation times (*t_x_*–*t_y_*). The plotting of mean gray values of every time‐lapse acquisition in the area of contraction additionally confirmed the differentiation into contracting cardiomyocytes, as can be seen in the time‐lapse movie (Movie S4, Supporting Information). Due to the heterogeneous beating pattern of spontaneously differentiating EBs (Figure S5 and Movie S5, Supporting Information), a more profound analysis of beating frequencies, amplitude and shape is not performed in the EST assay.

**Figure 6:**
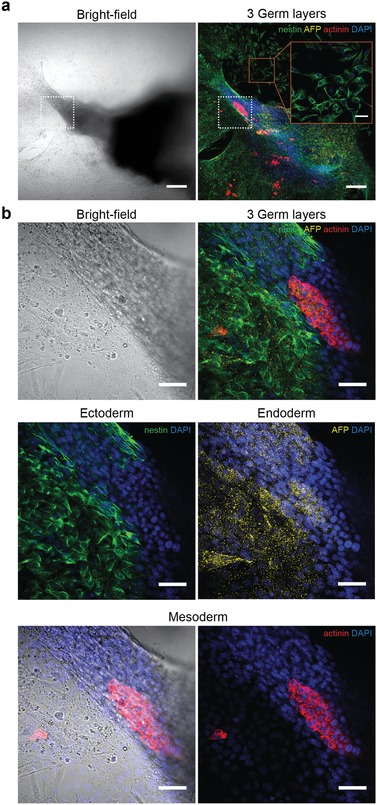
Immunofluorescent characterization of embryoid‐body differentiation. a) Bright‐field and confocal image of a spread‐out EB at 10× magnification, showing the spatial organization of the three germ layers at day 10. Ectodermal cells were stained with the neural‐progenitor marker nestin (green), endodermal cells with the carrier molecule alpha‐feto protein (AFP, yellow), and mesodermal cells with sarcomeric alpha actinin (red). The nuclear stain DAPI (blue) demonstrates the localization of the cells. A magnified area on the substrate surface (red frame) shows a monolayer of spread neural progenitor cells (scale bar: 50 µm). The area of contraction is indicated with a white frame. Scale bar: 200 µm. b) Bright‐field and confocal images of the area of contraction, indicated with the white frame in (a), at 40× magnification. Ectodermal cells, found at the periphery of the tissue, spread over the surface and formed monolayers of early neural progenitor cells. The endodermal marker AFP is scattered over the EB outgrowth zone, showing highest accumulation in AFP‐producing cells. Mesodermal cells were found in the center region of the EB, overlapping with the region of contraction (Figure 5b) as demonstrated in the bright‐field overlay. Scale bar: 50 µm.

We assessed the capacity of EB lineage commitment into the three germ layers ectoderm, endoderm, and mesoderm by immunofluorescent staining (**Figure** **6**). The spatial organization of the germ layers in the EB outgrowth can be seen in Figure 6a. A stitched 10× bright‐field image of the entire EB is compared to a confocal image of the same microtissue, which shows markers for all three germ layers as well as nuclei stained by DAPI. The ectoderm was stained with the neural progenitor marker nestin, the endoderm with glycoprotein alpha‐fetoprotein (AFP), and the mesoderm with the bundling protein sarcomeric alpha actinin, which is expressed in both, skeletal and cardiac muscle.

We imaged the site of contraction (Figure 6a, white dashed frame) with a higher magnification of 40× (Figure 6b). Nestin was most abundant in the outermost cell layers of the microtissue that were spreading over the substrate. The carrier molecule AFP was scattered over the EB outgrowth zone and accumulated in AFP‐producing cells. In contrast, the actinin stain was highly localized in the center of the EB.

By comparing the images obtained from the immunostainings to the functional readout of beating cardiomyocytes, we observed overlaps of actinin‐positive regions with areas of spontaneous contraction. An example for this correlation is demonstrated in Figure 5b, Movie S4 (Supporting Information), and Figure 6, which shows the same EB. The fact that the regions of spontaneous contractions in the morphological analysis matched with the regions of actinin staining verified the presence of cardiac muscle cells, as skeletal muscle cells do not show spontaneous contractions.

### 2.4 Bioactivation of Cyclophosphamide in the metaEST

**Figure 7:**
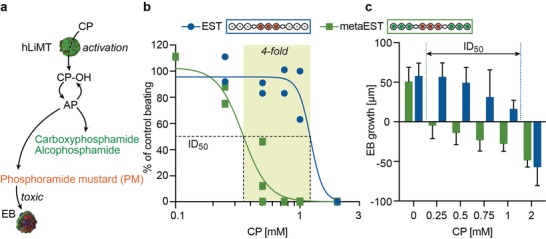
Bioactivation of cyclophosphamide (CP) in the metaEST. a) Metabolization of CP. CP gets activated through the liver to form the unstable intermediate 4‐hydroxycylophosphamide (CP‐OH) and its tautomer aldophosphamide (AP). Further decomposition yields the inactive metabolites carboxyphosphamide and alcophosphamide (green) and the toxic metabolite phosphoramide mustard (PM, red), which crosslinks DNA and produces embryotoxic effects. b) Concentration‐response curves of metaEST (green) and EST (blue) based on the morphological yes/no readout indicating the presence or absence of beating cardiomyocytes. Six concentrations of CP were tested in a dose range of 0–2 × 10^−3^ m (*x*‐axis). The *y*‐axis represents the percentage of wells in which beating areas were observed, normalized to beating areas in the control of 0 × 10^−3^ m CP. The ID50 concentration was (0.3 ± 0.03) × 10^−3^ m in the metaEST (*R* ^2^ = 0.96) and (1.2 ± 0.3) × 10^−3^ m in the EST (*R* ^2^ = 0.92), so that a fourfold lower ID50 value was found with the metaEST. EST: *n* = 17–24 EBs per condition in two individual experiments, metaEST: *n* = 7–31 EBs per condition in three individual experiments (Figure S6, Supporting Information). c) Change in EB diameter between day 3 and day 5 in the metaEST (green) and EST (blue). The dose‐dependent inhibition of growth correlates to the ID50 concentrations in (b); mean ± SD, *n* = 9–12 EBs per condition.

The embryotoxic prodrug cyclophosphamide is an alkylating agent, which interferes in mesodermal differentiation and causes severe congenital deformities or even death. The effect of CP depends on a biotransformation through the liver to form both, active and inactive metabolites (**Figure** **7**a). In a first step, CP gets metabolically activated through the liver to form 4‐hydroxycyclophosphamide (CP‐OH) and its tautomer aldophosphamide (AP).^42^ AP further reacts to yield the inactive metabolites alcophosphamide and carboxyphosphamide as well as the unstable intermediate phosphoramide mustard (PM), which crosslinks DNA and ultimately produces embryotoxic effects. We could detect the hLiMT‐mediated transformation of CP into alcophosphamide and carboxyphosphamide by UPLC‐MS analysis of snap‐frozen supernatant medium, whereas PM was not detectable due to its instable character and very short half‐life time of only a few minutes (Figure S6, Supporting Information).^43^ Degradation occurring during sampling, storage and sample processing would require to use analytical procedures, such as the inclusion of trapping agents or immediate stabilization of the analyte in the culture medium, in order to achieve reliable measurements.^44^

We then investigated the embryotoxic effect of PM in the metaEST experiment in coculture with hLiMTs by counting the number of wells, which displayed beating cardiomyocytes, and normalized the counts to those obtained under control condition. We performed the metaEST with CP in a dose range of 0–2 × 10^−3^ m and compared the results to those of the conventional assay without metabolic activation capacity (EST) that has also been executed in our platform, and the results of which are shown in Figure 7b and Figure S7 (Supporting Information). A clear shift toward lower ID50‐values was observed in the metaEST. Whilst the ID50 value in the conventional EST was determined to be 1.2 ± 0.3 × 10^−3^ m, the metaEST yielded a fourfold lower value of 0.3 ± 0.03 × 10^−3^ m. The decreased ID50 concentration in the multitissue configuration can, in our opinion, be attributed to the presence of instable embryotoxic metabolites, such as PM, generated by liver‐mediated biotransformation. The viability and functionality of the hLiMTs was maintained up to concentrations of 1 × 10^−3^ m CP, whereas 2 × 10^−3^ m CP caused hepatotoxic effects (Figure S2, Supporting Information). The dose‐dependent inhibition of EB differentiation is also reflected in the development of the EB diameters between day 3 and 5, shown in Figure 7c. With increasing CP concentration, a reduction in EB growth or even shrinkage was observed. This growth reduction or shrinkage is due to compaction of the microtissues, as well as cell death and disintegration of the EBs at high CP concentrations. Again, a clear difference in the results of metaEST and the conventional EST was observed. In this morphological analysis, the concentration at which the transition from tissue growth to tissue shrinkage occurred, correlated with the ID50 concentration obtained from the optical assessment of beating cardiomyocytes.

**Figure 8:**
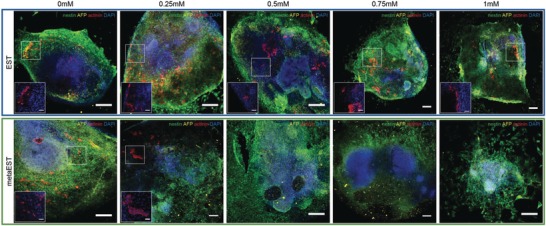
Immunofluorescent staining of spread‐out EBs treated with CP. Confocal images of spread EB at day 10 at 10× magnification (scale bar: 200 µm) showing dose‐dependent differentiation into mesodermal cells (red) in the EST and metaEST assay. Endodermal (yellow) and ectodermal (green) cells developed under all conditions. DAPI (blue) was used to counterstain the nuclei. Magnified areas of the EBs in the insets show mesodermal cells at 40× magnification (scale bar: 50 µm).

In order to complement the morphological endpoint assessment, immunofluorescent staining for the three germ layers was used as additional readout, as can be seen in **Figure** **8**. We observed a loss of mesodermal lineage expression with increasing concentrations of CP. The loss of mesodermal lineage expression in metaEST and EST correlates with the ID50 concentrations obtained from the morphological readouts (Figure 7b,c). CP was not found to have an effect on ectodermal and endodermal lineage commitment. Cytotoxic effects could be observed at CP concentrations exceeding the ID50 value, which resulted in reduced spreading and attachment of the cells to the substrate, shown in Figure S8 (Supporting Information).

Additionally, we confirmed the relevance of a coculture system with constant medium exchange in a static control experiment. Figure S9 (Supporting Information) shows the setup, which is the same as for the metaEST, however, with the decisive difference that the chip was not tilted so that no gravity‐driven flow through the connecting channels and chip was induced. hLiMTs and EBs were both present in the system. Due to the absence of induced flow, metabolite exchange between the compartments could only happen through diffusion through the channels. Under this static condition, we observed beating cardiomyocytes and mesodermal differentiation at day 10 for concentrations of up to 1 × 10^−3^ m CP. This result deviates from that obtained with a perfused system, in which inhibition of spreading and differentiation was evident for 1 × 10^−3^ m CP (Figures 7 and 8). This finding demonstrates that diffusion‐limited transport seems not to be sufficient to capture the effects of unstable intermediates and short‐lived metabolites, such as PM, on developmental processes in the EST.

## 3 Discussion

Advancing developmental in vitro assays toward physiologically more relevant model systems is of fundamental importance for a reliable characterization of potential drug candidates. Especially the inclusion of metabolic competence constitutes a decisive step toward better mimicking in vivo conditions and realizing more complex drug toxicity testing with respect to early embryonic development. In this work, we augment the well‐established EST by including the metabolic competence of the liver in order to take into account the potential biotransformation of compounds, which is essential to understand and faithfully represent a drug's mode of action.

The metaEST assay has been implemented as a robust coculture system of EBs and 3D primary hLiMTs in microfluidic HDNs. We integrated the entire coculture system in a single microfluidic device and, thereby, eliminated manual transfer steps between different culture substrates, which were necessary in the classical EST assay.^14^ For assessing metabolic effects already in an early stage of embryonic development, a single platform with fluidically interconnected hanging drops is key. Hanging‐drops are needed to enable the in situ formation, growth and subsequent spreading of EBs using a single substrate. At the same time, a direct and constant interaction with hLiMTs can only be realized by fluidically interconnecting individual hanging‐drop compartments from the start of the assay.

In particular, both 3D tissue models were cultured in very close proximity to each other to enable constant intertissue interaction through liver‐generated metabolites. The immediate transport and fast exchange of metabolites was further supported by gravity‐driven flow, which was realized through continuous tilting of the HDN. Constant perfusion is a prerequisite to also detect transiently stable or highly reactive metabolites, which are hardly detectable in a static system (Figure S9, Supporting Information). The continuous cocultivation in the same medium constitutes a central advantage over currently used static preincubation systems, which rely on the use of preincubated supernatant from a metabolically competent system.^25^, ^27^ The preincubation approach neglects the effect of nutrient depletion, which may significantly contribute to the experimental outcome (Figure S10, Supporting Information).

While keeping technological complexity at a minimum, we increased the relevance of the metaEST assay by integrating a primary human 3D liver model. The viability and functionality of the hLiMTs was maintained over more than 10 d on chip, which renders the approach suitable for long‐term studies, in particular developmental toxicity tests. Further, the included hLiMTs were capable of metabolizing the prodrug cyclophosphamide, which has been used as model compound in the metaEST assay. We demonstrated the undisturbed development of EBs in coculture with hLiMTs through their capacity to differentiate into all three germ layers in our microfluidic platform. Additionally, the differentiation into functional cardiomyocytes was confirmed by the presence of beating areas in the EB, which served as a simple readout of the assay. Combining both tissue models in the metaEST, yielded four‐times lower ID50 concentrations compared to the conventional EST with isolated EBs.

Previous studies that applied CP in the conventional validated EST assay without metabolic competence yielded ID50 concentrations of 1.3 × 10^−3^ 27 and 1.4 × 10^−3^ m 25 CP. These results compare well to the ID50 concentration of 1.2 × 10^−3^ m that has been measured in our system in the absence of liver MTs and serve as proof that the platform does not have an influence on the obtained results. Another study that includes metabolic features in an EST was realized by preincubating CP with a 2D layer of primary hepatocytes,^27^ and the authors also measured a lower ID50 concentration of 0.02 × 10^−3^ m upon biotransformation in comparison to ID50‐values obtained with a conventional EST assay. However, it is not possible to draw a direct comparison of the ID50 concentrations obtained by our and this 2D preincubation system, as 2D and 3D cell arrangements behave differently and as a reproduction of metabolic effects through preincubation and pipetting steps is limited.

A system offering the possibility to realize direct cocultures under constant perfusion may prove pivotal to detect toxic effects of transiently stable and short‐lived metabolites in an effort to better mimic physiological conditions and to obtain more meaningful compound testing results. Systemic effects of metabolic processes that may have been overlooked in static and monoculture assays need to be assessed.

Finally, we observed in our experiments a correlation between mesodermal lineage expression and the functional readout of beating cardiomyocytes so that such features could be used as a complementary readout to investigate a drug's or compounds toxicity or mechanism of action in more detail.

In summary, we developed a straightforward approach to combine modular 3D cell culture models in a simple and highly flexible platform, which could complement the conventional EST assay in tiered testing strategies as a second and advanced screening tool to eliminate false negative drug candidates of a conventional EST. In such a setting, the use of murine embryonic stem cells and EBs in the metaEST allows for a direct comparison of the obtained results with those of the preceding conventional EST. On the other hand, the use of a human liver model in the metaEST would allow to mimic human metabolization as closely as possible. The use of mouse ESCs and EBs in the metaEST assay also offers the advantage of keeping the assay time short, which is beneficial for acceptance and usage in industrial test settings. Nevertheless, an interesting future challenge will be to include human‐derived EBs from induced pluripotent stem cells in the metaEST to further reduce high attrition rates in animal trials and to increase the predictive power of such assays.

## 4 Experimental Section

*Microfluidic Chip Fabrication*: Microfluidic chips were fabricated from a 3D printed master mold (Accura SL 5530, Protolabs, Feldkirchen, Germany) by using soft lithography. PDMS and curing agent (Sylgard 184, Dow Corning Corp., Midland, Michigan, USA) were mixed at a 10:1 (w/w) ratio, degassed and poured onto the master mold at a thickness of 3 mm. After curing for 2 h at 80 °C, the PDMS layer was peeled off the master mold and cut into individual microfluidic chips. Each PDMS chip was then bonded to a standard microscopy slide (25 mm x 75 mm, 1 mm thick) after oxygen plasma treatment (Harrick Plasma PDC‐002, Harrick Plasma, Ithaca, NY, USA).

*Cell Culture*: The mouse embryonic‐stem‐cell line ES‐D3 was provided by InSphero AG, Schlieren, Switzerland. Cells were maintained in high‐glucose DMEM medium with GlutaMax (Thermofisher Scientific, Reinach, Switzerland) supplemented with 15% (v/v) ES qualified FBS (Merck Millipore, Schaffhausen, Switzerland), 1% (v/v) nonessential amino acids (NEAA, Sigma‐Aldrich, Buchs, Switzerland), 1% (v/v) 2‐Mercaptoethanol 100× (BME, Merck Millipore, Schaffhausen, Switzerland), and 1% (v/v) penicillin and streptomycin 100× (Merck Millipore, Schaffhausen, Switzerland). 1000 U mL^−1^ murine leukemia inhibitory factor (LIF, Merck Millipore, Schaffhausen, Switzerland) was added directly into the culture flask, which was coated with 0.1% gelatin solution (Merck Millipore, Schaffhausen, Switzerland) to promote cell adhesion. Cells were cultured at 37 °C in a humidified atmosphere under 5% CO_2_ and passaged every 2–3 d. Subculturing was carried out according to routine protocols. Briefly, mESCs were digested with Trypsin, centrifuged (x 200 g, 2 min), resuspended in medium and split into a 0.1% gelatin‐coated culture flask with 1000 U mL^−1^ LIF.

Primary human liver microtissues (hLiMTs, multidonor hepatocytes, monoculture, InSphero, Schlieren, Switzerland) were cultured in GravityTRAP plates (InSphero, Schlieren, Switzerland) and maintained in Liver AF medium (InSphero, Schlieren, Switzerland), which was changed every 2–3 d. For the use of primary human liver cells (Bioreclamation IVT, Brussels, Belgium), consent was obtained from all donors. No information was provided which might identify the donor. The hLiMTs were used for experiments within one week after delivery.

The customized coculture medium contained advanced DMEM medium (Thermofisher Scientific, Reinach, Switzerland) enriched with 3.5% (v/v) primary hepatocyte maintenance supplement (Thermofisher Scientific, Reinach, Switzerland), 5% (v/v) FBS, 1% (v/v) NEAA, 1% (v/v) BME and 1% (v/v) penicillin and streptomycin 100×.

*Chip Preparation*: The microfluidic chips were cleaned with soap, water and isopropanol and dried using an air gun. The PDMS channel and drop structures were then rendered hydrophilic to facilitate liquid loading. Therefore, the PDMS structures were selectively plasma‐activated by covering the top part of the chip with a thin PDMS mask featuring small holes at the drop sites. Oxygen plasma could then only access the microfluidic channel and drop structures, while the top part of the rim remained hydrophobic to prevent liquid overflow.

The hydrophilic drop compartments were coated with 0.1% gelatin solution (Merck Millipore, Schaffhausen, Switzerland) in the EB compartments and with Biolipidure (NOF America Corporation, WhitePlains, NY, USA) in the hLiMT compartments and incubated at room temperature (RT) for 30 min. Biolipidure solution was withdrawn completely from the hLiMT compartments by using a vacuum pump, and these compartments of the chips were coated a second time with Biolipidure. After incubating for another 30 min, the coating was removed from both, EB and hLiMT compartments, and the chips were dried overnight under sterile conditions in the laminar‐flow hood.

*hLiMT Induction*: The medium of the hLiMTs was exchanged one day before the experiment with medium containing 0, 0.25, 0.5, 0.75, 1, and 2 × 10^−3^ m CP monohydrate ISOPAC (C7397‐1G, Sigma‐Aldrich, Buchs, Switzerland) in order to preinduce their metabolic activity. CP containing medium was always freshly prepared before use.

*MetaEST*: Chip loading at day 0 was carried out in two steps: i) hLiMT loading and ii) EB loading.

i) The hLiMT compartments of each chip were filled with medium containing the desired CP concentration (0–2 × 10^−3^ m) using a volume of 18.3 µL medium per drop compartment. Every chip was covered with a plastic lid (µ‐slide Angiogenesis, Ibidi, Basel, Switzerland) to prevent medium evaporation and was then placed in the incubator at 37 °C and 5% CO_2_ to warm up the medium. Afterward, hLiMTs were manually transferred from the GravityTRAP plate (InSphero, Schlieren, Switzerland) into the corresponding drop compartments. For that, hLiMTs were aspirated in 15 µL medium using a pipette. The microtissues sedimented to the bottom of the pipette tip and were then loaded into the chip by bringing the pipette tip into contact with the medium in the culture compartments without piston activation. After hLiMT loading, the plastic lid was properly inserted into the dedicated groove structure on the chip. The lid was only removed during chip loading and medium exchange. The chips were inserted into a chip holder (handling frame with high skirt, Microfluidic Chip Shop, Jena, Germany) in a hanging‐drop configuration, where each holder hosted four chips. The chips were kept in the incubator at 37 °C and 5% CO_2_ while preparing the stem cells.
ii) The mESCs were harvested from the culture flask and re‐suspended in medium containing the defined CP concentration in order to obtain 750 cells per drop. The chip holders were taken from the incubator and flipped upside‐down to obtain a standing‐drop configuration. 18.3 µL of cell suspension were pipetted into the corresponding EB compartments of the chip. The chip holder was then flipped to achieve a hanging‐drop configuration so that the mESCs could sediment to the liquid–air interface to form EBs. The chip holder was covered with a plastic lid (Chip lid Fluidic 854, Microfluidic Chip Shop, Jena, Germany) and then placed onto a 96‐well plate (NUNC Microwell 96F, Thermofisher Scientific, Reinach, Switzerland), which was filled with PBS. The setup was placed in an incubator at 37 °C and 5% CO_2_ in a horizontal position for 24 h to allow undisturbed EB formation.

After 24 h, the chip holder was taken from the incubator and flipped to obtain a standing‐drop configuration. The capillary stop valves between the hLiMT and EB compartments were opened by wetting the valves with medium to accommodate both tissue types in the same fluidic network and to establish the coculture. The chip setup was then flipped back to a hanging‐drop configuration and placed on a tilting stage (Multi Bio RS‐24, InSphero, Schlieren, Switzerland) and tilted by ± 2° with a tilting motion during 10 s, and a holding phase of the tilted position for 5 s to induce flow through the network and to enable intertissue communication and metabolite exchange.

At day 3 and 5, the chip setup was taken off the tilting device, and 70 µL of medium in each fluidic network were exchanged after flipping the chip holder to a standing‐drop configuration. The medium was carefully exchanged at the locations of the capillary valves. After medium exchange at day 5, the chips were carefully removed from the chip holder, flipped upside‐down and inserted again in a standing‐drop configuration. The setup was then placed on the tilting stage and tilted at ± 4° with a tilting motion during 10 s, and a holding of the tilted position for 5 s. Media exchanges were carried out at day 7, 8 and 9.

At day 10, the EB compartments were optically inspected for beating areas within the EB outgrowth zone. The wells in which beating cardiomyocytes were observed between day 7 and day 10 were counted for each condition. The percentage of beating cardiomyocytes per condition was normalized to the untreated control. For downstream analysis, hLiMTs were collected from the chip by pipetting and transferred to a GravityTrap plate (InSphero, Schlieren, Switzerland) for subsequent biochemical assays.

*EST with Preincubated Medium*: The hLiMTs were incubated in 50 µL coculture medium containing 0 × 10^−3^, 0.1 × 10^−3^, and 1 × 10^−3^ m CP in a GravityTrap plate (InSphero, Schlieren, Switzerland) for 72 h. The medium was sampled and stored at −20°. At day 0 of the EST, mESCs were suspended in the preincubated medium and seeded into a GravityPlus plate (InSphero, Schlieren, Switzerland) with a cell count of 750 cells per 40 µL drop. EBs were formed during 3 d in the incubator at 37 °C. At day 3, 50% of the preincubated medium was replenished. At day 5, EBs were transferred to a 96‐well plate (NUNC Microwell 96F, Thermofisher Scientific, Reinach, Switzerland) with a volume of 150 µL preincubated medium for EB spreading. At day 7, 50% of the preincubated medium was exchanged, and the readout of contracting cardiomyocytes was carried out at day 10.

*Immunofluorescent Staining*: EBs were fixed, and immunofluorescent staining was performed on‐chip for the three germ layers. EBs were washed in PBS and fixed with 4% paraformaldehyde (Thermofisher Scientific, Reinach, Switzerland) for 20 min at RT. The samples were washed three times in PBS and incubated in blocking solution, containing 10% normal donkey serum (NDS), 1% bovine serum albumin (BSA) and TritonX solution (Sigma‐Aldrich, Buchs, Switzerland). Primary antibodies against the mesodermal marker sarcomeric alpha actinin (Abcam, Cambridge, UK, ab137346, rabbit, 1:200), the endodermal marker alpha fetoprotein (R&D Systems, Minneapolis, Minnesota, USA, AF5369, goat, 1:200), and the ectodermal marker nestin (Abcam, Cambridge, UK, ab134017, chicken, 1:200) were added in reaction solution containing 3% NDS and 1% BSA and incubated at 4° overnight. After washing the samples three times in PBS, the MTs were incubated with the matching secondary antibodies: donkey‐anti‐rabbit (Invitrogen, Carlsbad, California, USA, A‐31572, 555 nm ,1:200) for the mesoderm, donkey‐anti‐goat (Abcam, Cambridge, UK, ab150131, 647 nm, 1:200) for the ectoderm, donkey‐anti‐chicken (Jackson Immunoresearch Laboratories, Cambridge, UK, 703‐535‐155, 488 nm ,1:200) for the endoderm. The nuclear stain DAPI (Thermofisher Scientific, Reinach, Swizterland, 1 mg mL^−1^, 62 248, 1:200) was added, and the cells were incubated in reaction solution containing 3% NDS and 1% BSA for 90 min in the dark at RT. After washing the samples again three times in PBS, the EBs were imaged on a microscope.

*Image Acquisition*: The MTs were monitored, and the optical readout at the end of each experiment was carried out on an inverted microscope (Nikon Ti‐E, Nikon, Egg, Switzerland) with a Nikon Plan Fluor 10x objective (NA 0.3, WD 16 mm) in bright‐field mode, using the Youscope software. Both microscopes were equipped with a stage top incubator, connected to a CO_2_ (5%), humidity (95%) and temperature (37 °C) regulator.

To visualize the germ layers of the immunostained EBs, imaging was performed with a Nikon A1 confocal microscope (Nikon, Egg, Switzerland) with a Nikon Plan Fluor 10x objective (NA 0.3, WD 16 mm) and a Nikon Plan Fluor ELWD 40x objective (NA 0.6, WD 3.7–2.7 mm), operated with the NIS‐Elements software (Nikon, Egg, Switzerland).

*Image Analysis*: Microscopy images were analyzed with ImageJ. To visualize the area of contraction, an image subtraction (differential) of two images of the time‐lapse acquisition (contraction‐relaxation; *t_x_*–*t_y_*) was performed. The profile of the contracting area was generated by plotting the mean gray value of every time‐lapse image in the ROI (*z*‐plot). These intensity values were further normalized to the highest value using Matlab.

*ATP Assay*: The ATP content was evaluated by using the CellTiter‐Glo Luminescent Cell Viability Assay (Promega, Dübendorf, Switzerland) following the manufacturer's protocol. The luminescence signal was detected with a microplate reader (Infinite M200pro, TECAN, Männedorf, Switzerland).

*Albumin ELISA*: The albumin content of the collected supernatants was determined by using an enzyme‐linked immunosorbent assay (ELISA, Bethyl Laboratories, Montgomery, Texas, USA) according to manufacturer's instructions. Reference serum standards were used for the calibration curve to calculate the albumin concentration of the samples. All samples were analyzed in duplicates. The absorption was measured at 450 nm with a microplate reader (Infinite M200 Pro, TECAN, Männedorf, Switzerland).

*CYP Activity*: The hLiMTs were incubated in 0 × 10^−3^, 0.25 × 10^−3^, 0.5 × 10^−3^, 0.75 × 10^−3^, 1 × 10^−3^, and 2 × 10^−3^ m CP in the presence of 100 × 10^−6^ m Bupropion and 3 × 10^−6^ m Midazolam for 24 h in the liver‐specific medium “Liver TOX” (InSphero, Schlieren, Switzerland). CYP activity was measured at day 0 and 10 of the assay using 3 hLiMTs per condition and time point. After 24 h of incubation, 50 µL of the medium was sampled from each hLiMT and snap‐frozen on dry ice. The sample plate was kept at −80° and the supernatant was tested for substrate conversion by LC‐MS by Pharmacelsus GmbH, Saarbrücken, Germany.

*Mass Spectrometry*: The hLiMTs were incubated in 1 × 10^−3^ m CP in a GravityTrap plate (InSphero, Schlieren, Switzerland) in 50 µL coculture medium. Samples were taken after 6, 12, 24, 48, and 72 h from six individual MTs per condition. For this, 5 µL supernatant were sampled from each well and snap‐frozen into 45 µL coculture medium on dry ice. The sample plate was kept at −80° and sent for analysis on dry ice to Admescope, Oulu, Finland.

*Statistical Analysis*: All error bars represent s.d. if not specified otherwise. If no s.d. is indicated, the error is smaller than the size of the symbol, which represents the mean value. The half‐maximal inhibitory concentration (ID50) was determined by applying a nonlinear regression analysis. A three‐parameter logistic function was fitted to the raw data.

## Conflict of Interest

O.F. is working for InSphero AG commercializing 3D microtissues and organ‐on‐chip solutions.

## Supporting information

Supplementary

Supplementary

Supplementary

Supplementary

Supplementary

Supplementary
