# Supplementary material for: Microfluidic Multitissue Platform for Advanced Embryotoxicity Testing In Vitro
Source: Adv Sci (Weinh). 2019 Apr 29;6(13):1900294. doi: 10.1002/advs.201900294 (PMC6662399; doi:10.1002/advs.201900294)
Supplement: Supplementary file 1 — Supplementary [file ADVS-6-1900294-s002.pdf]

Copyright WILEY-VCH Verlag GmbH & Co. KGaA, 69469 Weinheim, Germany, 2019.

ADVANCED SCIENCE
Open Access

Supporting Information

for *Adv. Sci.,* DOI: 10.1002/advs.201900294

Microfluidic Multitissue Platform for Advanced Embryotoxicity Testing In Vitro

Julia Alicia Boos, Patrick Mark Misun, Astrid Michlmayr, Andreas Hierlemann, and Olivier Frey**

Copyright WILEY-VCH Verlag GmbH & Co. KGaA, 69469 Weinheim, Germany, 2019.

Supporting Information

Microfluidic multi-tissue platform for advanced embryotoxicity testing in vitro

Julia Alicia Boos, Patrick Mark Misun, Astrid Michlmayr, Andreas Hierlemann and Olivier Frey**

Table S1: Development of the co-culture medium. Different basal media, supplemented with varying concentrations of FBS and additional culture supplements were tested. NEAA, 2-ME and Pen/Strep were always supplemented with 1% (v/v). PHM was supplemented with 3.5% (v/v). EBs were examined according to their capability to form 3D microtissues (formation) within 3 days and to differentiate into contracting cardiomyocytes (beating) at day 10. hLiMTs were tested according to their viability (ATP) and functionality (Albumin). The albumin secretion of samples exhibiting poor ATP values or unsuccessful EB formation or beating was not further analyzed. The 'metaEST medium' was used for co-culture experiments and is highlighted in the table in yellow. Acronyms: fetal bovine serum (FBS), Dulbecco's modified Eagle's Medium (DMEM), Knockout serum replacement (KSR), non-essential amino acids (NEAA), 2-Mercaptoethanol (2-ME), Penicillin-Streptomycin (Pen/Strep), primary hepatocyte maintenance supplements (PHM).

Co-culture medium			EB		hLiMT	
Basal medium	FBS	Supplements	Formation	Beating	ATP [pmol x MT^{-1}]	Albumin [ng x MT^{-1} x d^{-1}]
Liver TOX a)	0%	-	no	no	16.97	14.33
William's E	0%	-	no	no	14.09	-
DMEM (high glucose, GlutaMax)	0%	NEAA, 2-ME, Pen/Strep	no	no	15.47	-
Liver TOX	5%	-	yes	no	15.28	16.72
advanced DMEM	5%	NEAA, 2-ME, Pen/Strep, PHM	yes	yes	23.48	13.85
advanced DMEM	5%	NEAA, 2-ME, Pen/Strep	yes	yes	16.28	-
DMEM (high glucose, GlutaMax)	10%	NEAA, 2-ME, Pen/Strep	yes	partially	5.47	-
DMEM (high glucose, GlutaMax)	15% (KSR)	NEAA, 2-ME, Pen/Strep	yes	no	11.31	-
DMEM (high glucose, GlutaMax) b)	15%	NEAA, 2-ME, Pen/Strep	yes	yes	4.76	-

a) Optimized medium for hLiMT toxicological studies, commercially available through InSphero AG, Schlieren, Switzerland.
b) Medium composition used in the validated EST assay.

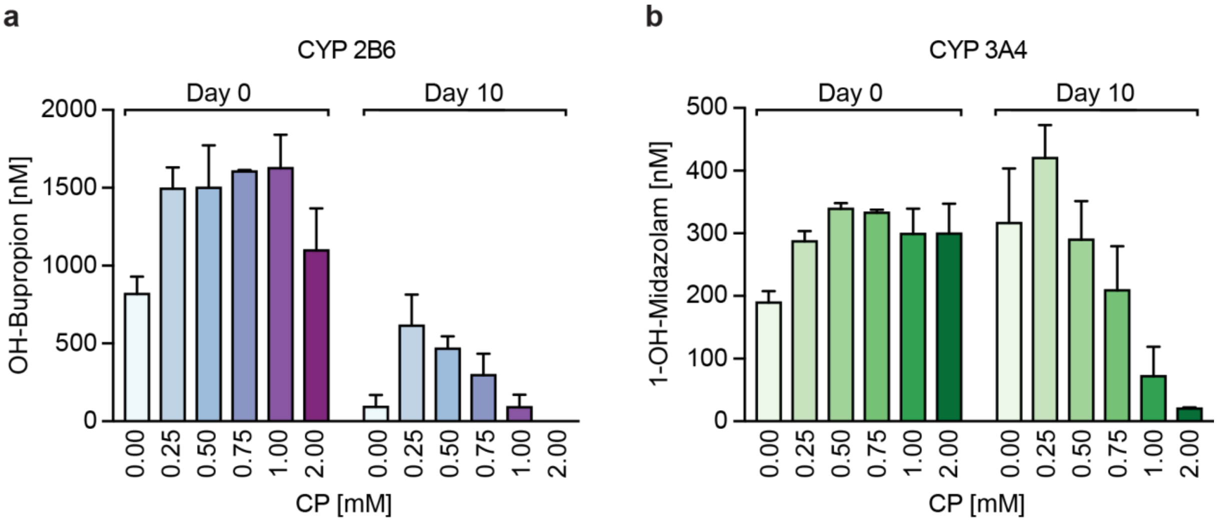

Figure S1. Basal and inducible CYP activities of hLiMTs. (a) CYP2B6 and **(b)** CYP3A4 activity after incubation of hLiMTs with CYP substrates Bupropion (100 µM) and Midazolam (3 µM). CYP activity was measured at day 0 and day 10 of the assay after induction with CP within a dose-range of 0-2 mM. hLiMTs were induced with CP 24 h prior to day 0 and constantly exposed to CP during the assay. The supernatant of hLiMTs was tested for substrate conversion by LC-MS; mean ± SD, *n* = 3 hLiMTs per condition.

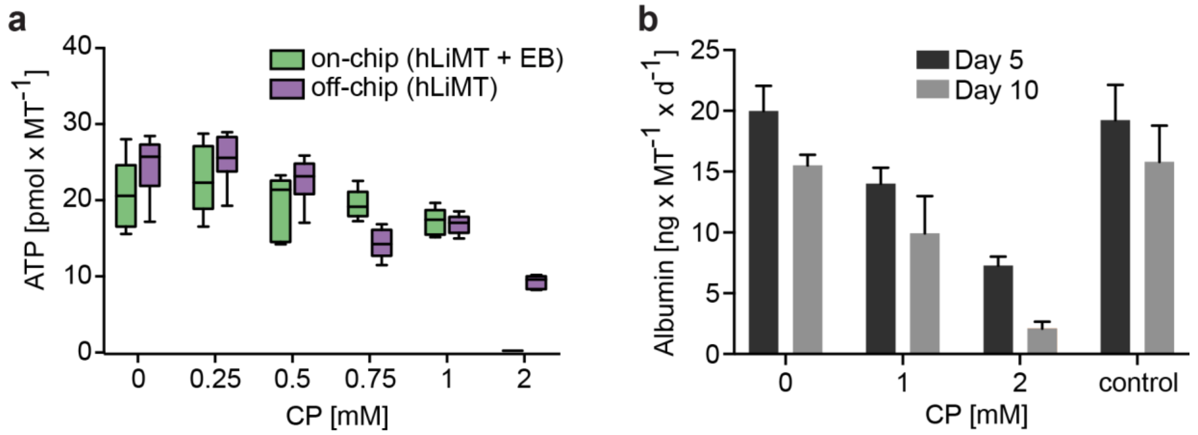

Figure S2. ATP content and Albumin secretion of hLiMTs treated with CP. (a) ATP content at day 10 for the tested CP concentrations (0-2 mM). ATP of hLiMTs was measured after co-culture experiments with EBs on-chip and in a static well plate without EBs; mean ± SD, *n* = 5-6 hLiMTs. **(b)** Albumin production at day 5 and 10 for CP concentrations of 0, 1 and 2 mM and comparison to hLiMT-specific control medium. Albumin was measured in a static well plate without EBs; mean ± SD, *n* = 6 hLiMTs.

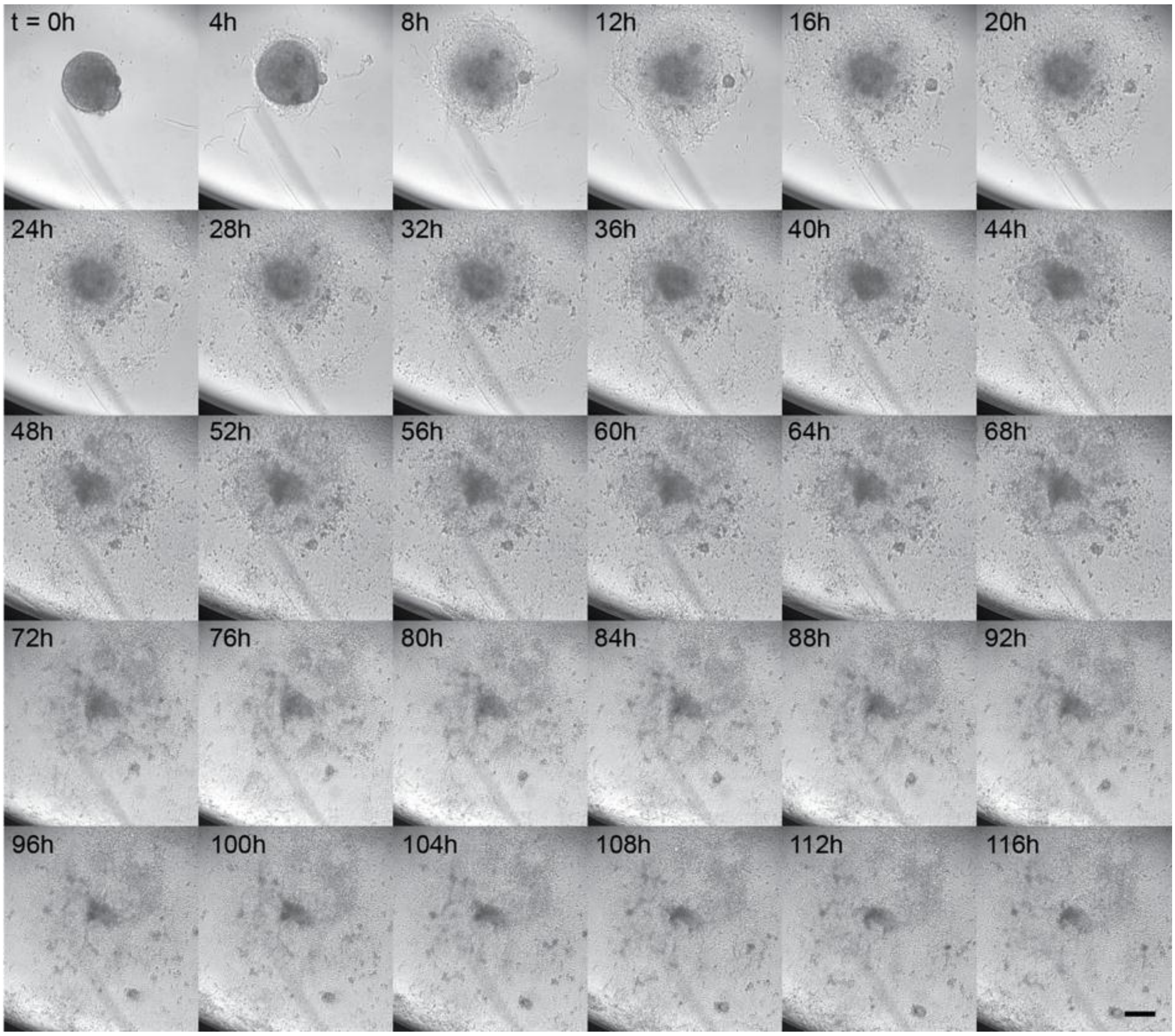

Figure S3. Spreading of EB under static conditions. Time-lapse, bright-field photographs of the spreading process of an embryoid body under static conditions without flow. The photographs were taken from day 5 to day 10 of the assay. Scale bar: 200 µm.

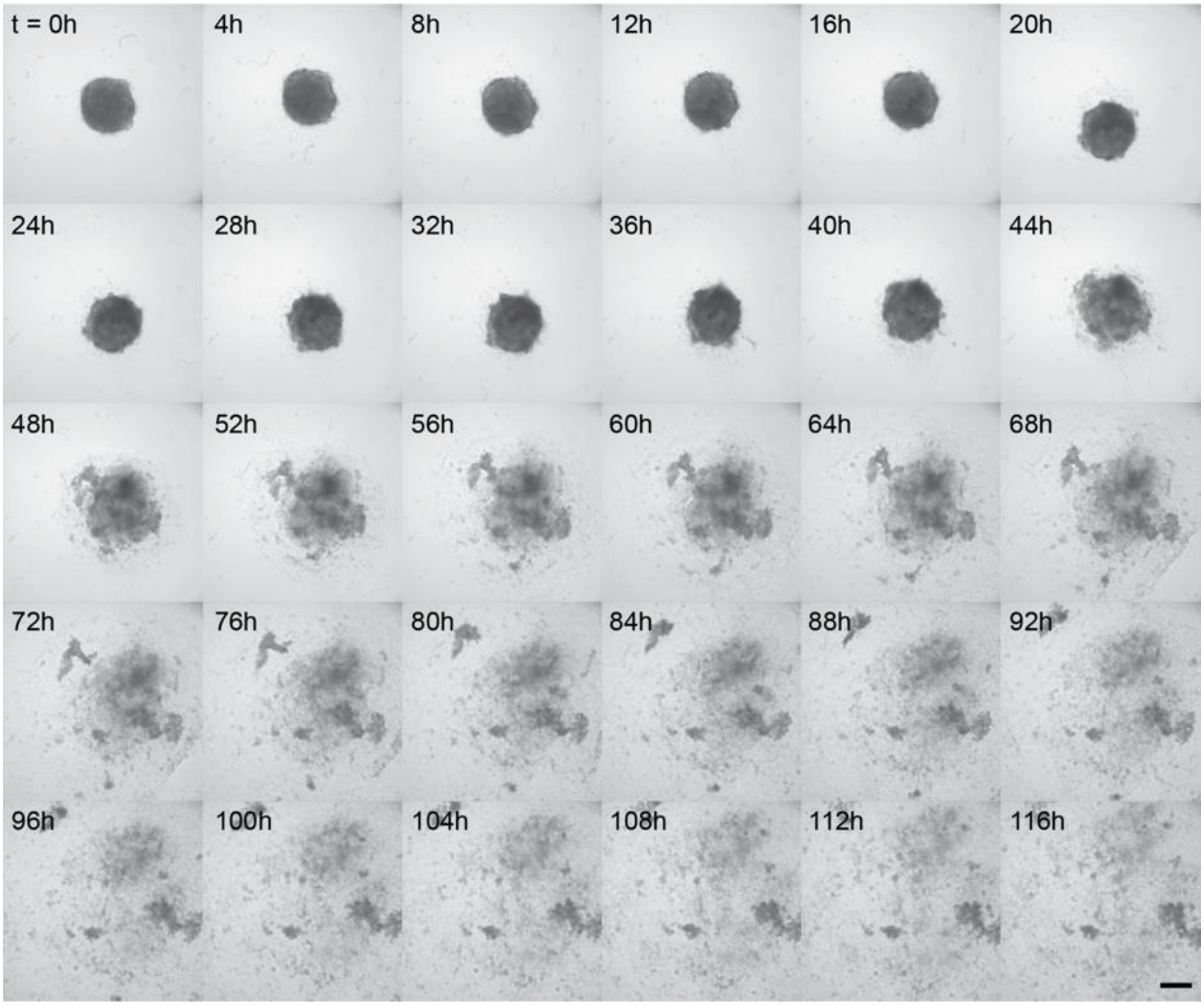

Figure S4. Spreading of EB under flow conditions. Time-lapse, bright-field photographs of the spreading process of an embryoid body under flow conditions. The photographs were taken from day 5 to day 10 of the assay. Scale bar: 200 µm.

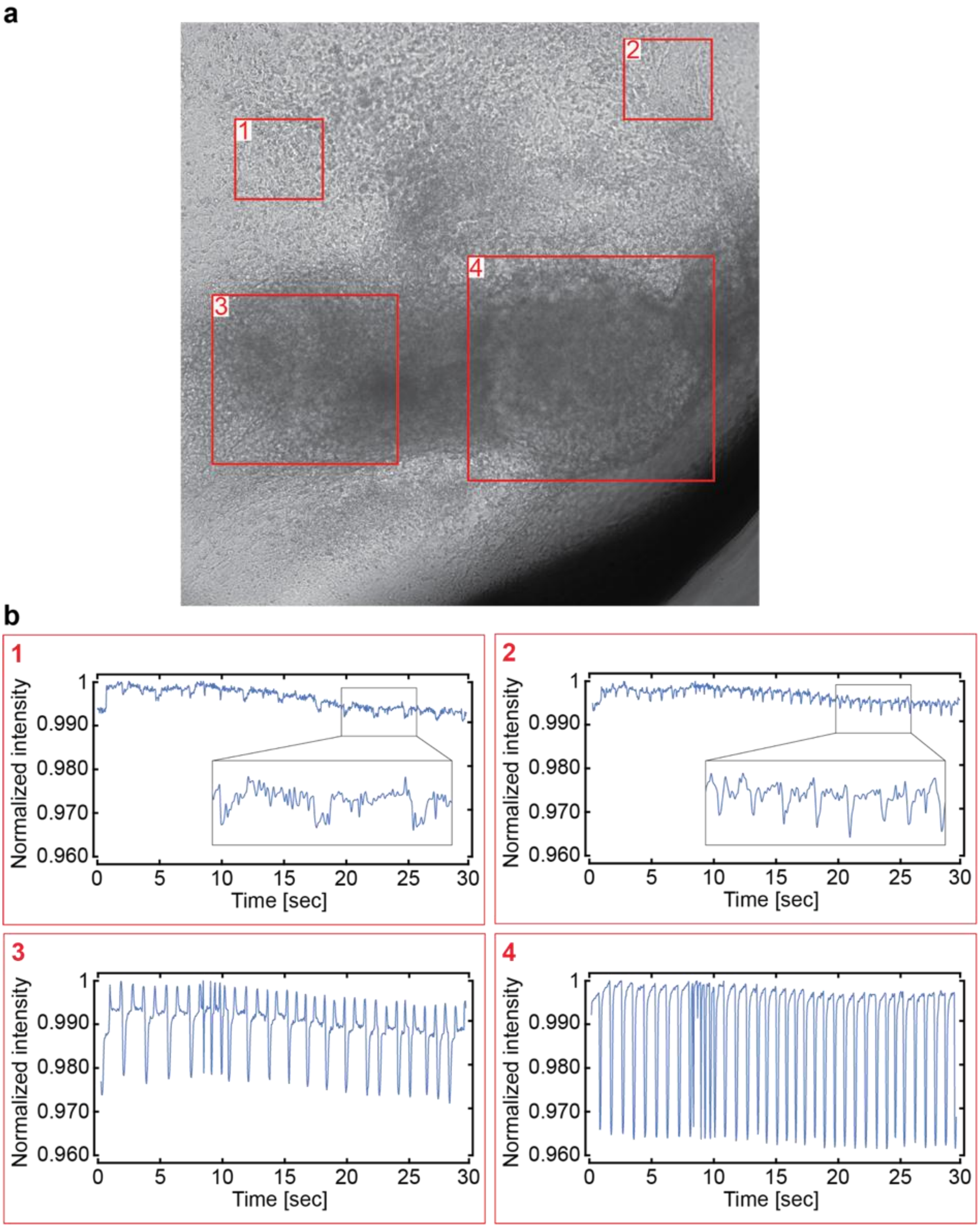

Figure S5. Heterogeneous beating pattern within a spread-out EB. (a) Bright-field photograph of a beating EB at day 10 under mono-culture conditions. The areas of contraction are indicated by red frames (1-4, see Movie S5). **(b)** The intensity profile of mean grey values of every time-lapse acquisition in the areas of contraction shows the beating intervals and frequency during 30 sec. Beating patterns within a single microtissue vary in intensity, strength, and size of beating areas. Scale bar: 200 µm.

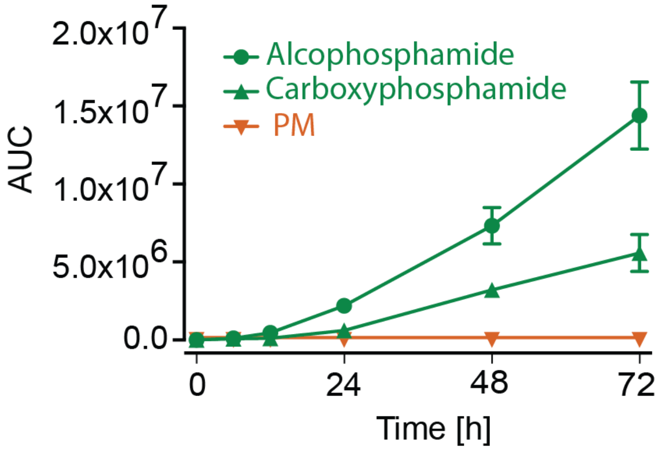

Figure S6. Metabolization of CP by hLiMTs. UPLC-MS analysis of snap-frozen supernatant of hLiMTs cultured with 1 mM CP. Samples were taken at 0 h, 6 h, 12 h, 24 h, 48 h and 72 h. The area under the curve (AUC) shows detection of inactive and active metabolites. The inactive metabolites alcophosphamide and carboxyphosphamide (green) were initially detected after 6 h and accumulated over three days due to their chemical stability. The toxic metabolite phosphoramide mustard (PM, red) could not be detected due to its short half-life time; mean ± SD, n = 3 hLiMTs per time point.

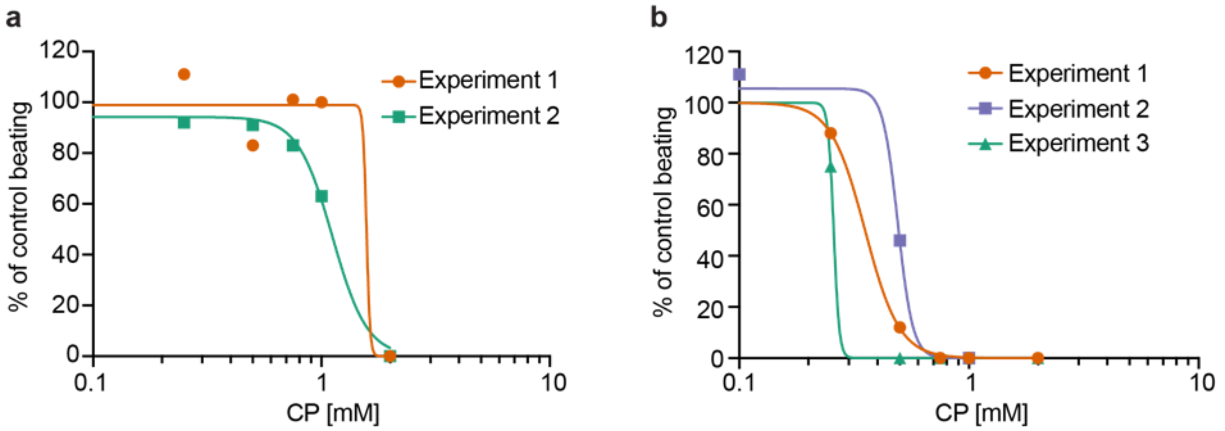

Figure S7. Concentration-response curves of EST and metaEST upon dosage of cyclophosphamide (CP). Concentration-response curves of the individual experiments in **(a)** the EST and **(b)** the metaEST assay. Concentrations of CP were tested in a dose range of 0-2 mM (x-axis). The y-axis represents the percentage of beating EBs normalized to that of the control of 0 mM CP. EST: *n* = 7-12 EBs per condition and experiment, metaEST: *n* =4-12 EBs per condition and experiment.

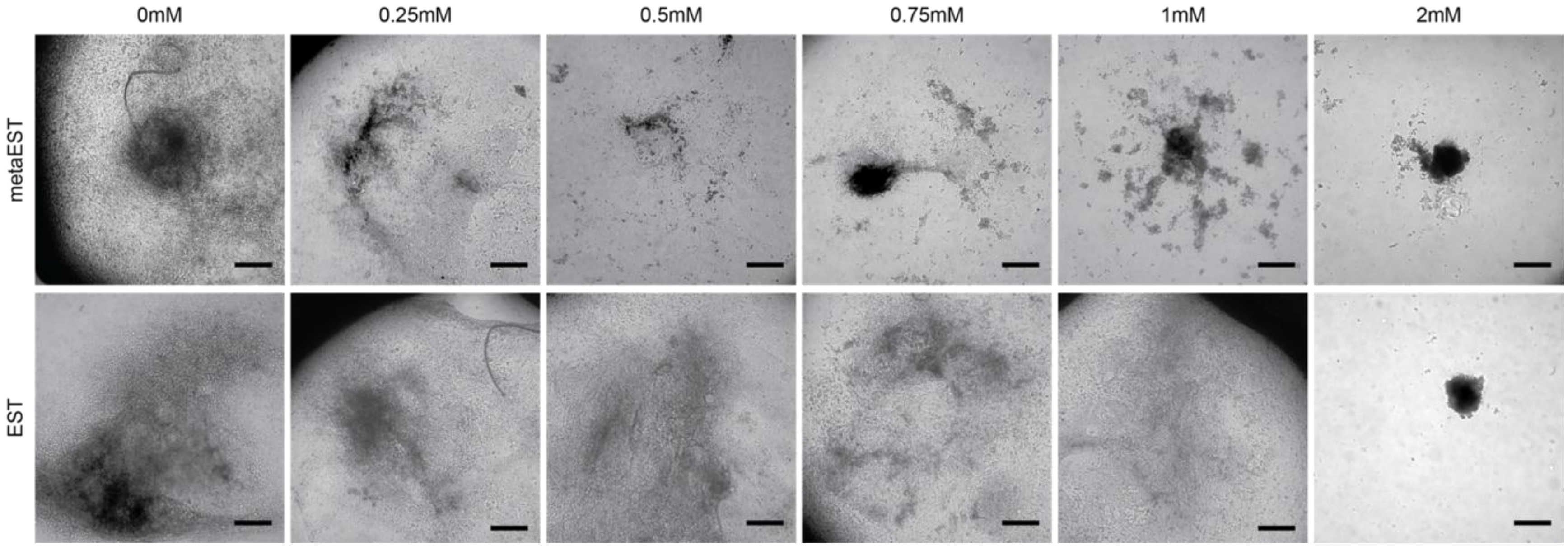

Figure S8. Spread-out EBs at day 10 treated with CP. Bright-field images of spread-out EBs at day 10 at 10x magnification of the metaEST and EST assay. EBs were treated with CP in a dose-range of 0-2 mM, showing a dose-dependent inhibition of spreading and growth; scale bar: 200 µm.

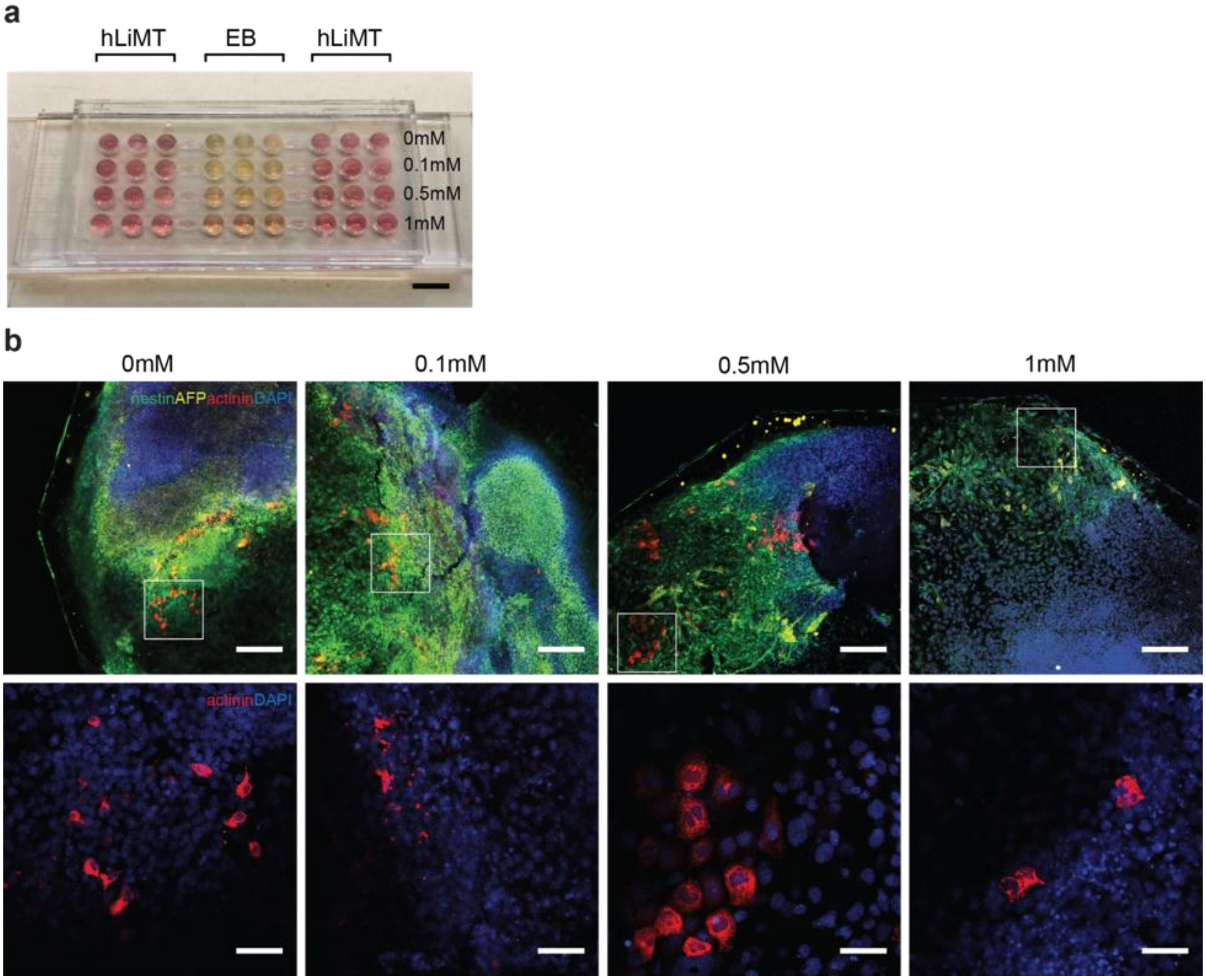

Figure S9. Static metaEST in the absence of gravity-driven flow (no tilting of the hanging-drop system). (a) Photograph of microfluidic chip. Each row has been used for one CP concentration (0-1 mM). EBs were cultured in the central drops, hLiMTs were cultured at the left and right side. The change in drop color correlates to the medium consumption in the tissue compartments: at higher CP concentration cell proliferation is reduced; scale bar: 5 mm. **(b)** Confocal images of spread-out EBs at day 10 at 10x magnification (scale bar: 200 µm) showing the three germ layers ectoderm (green), mesoderm (red), and endoderm (yellow), as well as the nuclei, stained with DAPI (blue). Magnified areas in the EBs in the bottom row show mesodermal cells at 40x magnification (scale bar: 50 µm).

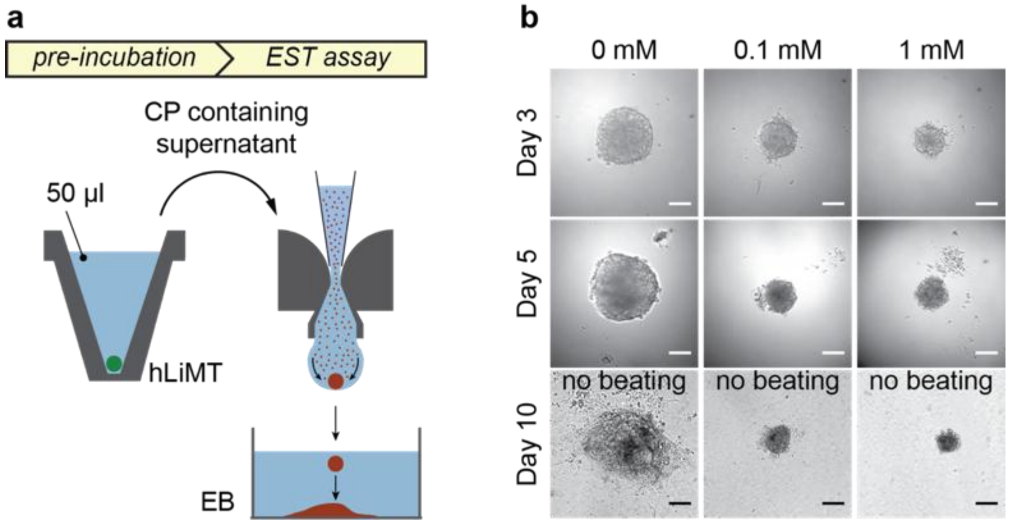

Figure S10. Bio-activation EST with pre-incubated medium. (a) Schematic representation of the experimental procedure. MetaEST medium with 0, 0.1 and 1 mM CP was pre-incubated with an hLiMT in a well plate, and the supernatant was harvested after 48 h. Pre-incubation time was based on CP metabolization by the hLiMTs (Figure S6) to generate a sufficiently high concentration of metabolites in the supernatant. Each well contained one hLiMT in a volume of 50 µl. The CP-containing supernatant was used to perform the validated standard EST assay. **(b)** Microscopic images of EBs at days 3, 5, and 10. No beating cardiomyocytes were observed for any of the three conditions at day 10. 0 mM CP: n=12; 0.1 mM and 1 mM: n=5; scale bar: 100 µm.

Movie S1: Time-lapse bright-field photographs (acquisition interval 30 min, 5 fps) of embryoid-body formation on-chip. The initial cell concentration was 750 cells per drop. Scale bar: 200 µm.

Movie S2: Time-lapse bright-field photographs (acquisition interval 30 min, 2 fps) of the spreading process of an embryoid body under static conditions without flow over a duration of 5 days. Scale bar: 200 µm.

Movie S3: Time-lapse bright-field photographs (acquisition interval 30 min, 2 fps) of the spreading process of an embryoid body under flow conditions over a duration of 5 days. Scale bar: 200 µm.

Movie S4: Functional readout of beating cardiomyocytes in the spread-out EB under co-culture conditions. The beating was captured by constant recording during 30 sec (24 fps). The intensity-profile of mean grey values of every time-lapse acquisition in the area of contraction shows the beating intervals and frequency. Scale bar: 200 µm.

Movie S5: Heterogeneous beating pattern of a spread-out EB under mono-culture conditions. The areas of beating cardiomyocytes are indicated by red frames (see Figure S5). Functional readout of beating cardiomyocytes in the spread-out EB under mono-culture conditions. Beating patterns within a single microtissue can vary in intensity, strength, and size of beating area. The beating was captured by constant recording during 30 sec (24 fps). Scale bar: 200 µm.
